# Molecular insights into the role of genetic determinants of congenital hypothyroidism

**DOI:** 10.5808/gi.21034

**Published:** 2021-09-30

**Authors:** Yedukondalu Kollati, Radha Rama Devi Akella, Shaik Mohammad Naushad, Rajesh K. Patel, G. Bhanuprakash Reddy, Vijaya R. Dirisala

**Affiliations:** 1 Department of Biotechnology, Vignan's University, Vadlamudi, Guntur, Andhra Pradesh 522213, India; 2 Department of Genetics, Rainbow Children's Hospital, Banjara Hills, Hyderabad, Telangana 500009, India; 3 Department of Biochemical Genetics and Pharmacogenomics, Sandor Speciality Diagnostics Pvt. Ltd, Banjara Hills, Hyderabad, Telangana 500034, India; 4 Department of Genetics, Genetic Group of Gujarat Diagnostic Centre, Mehsana, Gujarat 384002, India; 5 Biochemistry Division, National Institute of Nutrition, Hyderabad, Telangana 500007, India

**Keywords:** congenital hypothyroidism, miR, *TG*, *TPO*, *TSHR*, 3′-UTR

## Abstract

In our previous studies, we have demonstrated the association of certain variants of the thyroid-stimulating hormone receptor (*TSHR*), thyroid peroxidase (*TPO*), and thyroglobulin (*TG*) genes with congenital hypothyroidism. Herein, we explored the mechanistic basis for this association using different *in silico* tools. The mRNA 3'-untranslated region (3'-UTR) plays key roles in gene expression at the post-transcriptional level. In *TSHR* variants (rs2268477, rs7144481, and rs17630128), the binding affinity of microRNAs (miRs) (hsa-miR-154-5p, hsa-miR-376a-2-5p, hsa-miR-3935, hsa-miR-4280, and hsa-miR-6858-3p) to the 3'-UTR is disrupted, affecting post-transcriptional gene regulation. TPO and TG are the two key proteins necessary for the biosynthesis of thyroid hormones in the presence of iodide and H_2_O_2_. Reduced stability of these proteins leads to aberrant biosynthesis of thyroid hormones. Compared to the wild-type TPO protein, the p.S398T variant was found to exhibit less stability and significant rearrangements of intra-atomic bonds affecting the stoichiometry and substrate binding (binding energies, ΔG of wild-type vs. mutant: ‒15 vs. ‒13.8 kcal/mol; and dissociation constant, K_d_ of wild-type vs. mutant: 7.2E^-12^ vs. 7.0E^-11^ M). The missense mutations p.G653D and p.R1999W on the TG protein showed altered ΔG (0.24 kcal/mol and 0.79 kcal/mol, respectively). In conclusion, an *in silico* analysis of *TSHR* genetic variants in the 3'-UTR showed that they alter the binding affinities of different miRs. The TPO protein structure and mutant protein complex (p.S398T) are less stable, with potentially deleterious effects. A structural and energy analysis showed that TG mutations (p.G653D and p.R1999W) reduce the stability of the TG protein and affect its structure-functional relationship.

## Introduction

Congenital hypothyroidism (CH) is one of the most common endocrine disorders, reported to occur in 1 in 3,000 to 4,000 newborns worldwide [1,2], and 1 in 1,100 in India [3]. On a worldwide basis, CH frequently results from iodine deficiency. Otherwise, CH is commonly caused by thyroid gland development defects, which can lead to thyroid dysgenesis (80%‒85%) [1]. The majority of these cases involve thyroid dysgenesis [4], agenesis (35%‒40%), ectopic tissue (30%‒45%), or hypoplasia (5%) [1]. Thyroid dysgenesis is caused by genes (thyroid transcription factor-1 [*TTF-1*], thyroid transcription factor-2 [*TTF-2*], and paired box 8 [*PAX-8*]) associated with syndromic CH and those causing non-syndromic CH (thyroid-stimulating hormone receptor [*TSHR*]) [5]. The remaining 15%-20% of cases are due to hereditary defects in the genes involved in the intermediary steps of biosynthesis in the thyroid, leading to dyshormonogenesis [6]. Thyroid dyshormonogenesis is associated with multiple genetic defects, including dual oxidases (*DUOXs*; *DUOX1* and *DUOX2*), and its maturation factors (*DUOXA1* and *DUOXA2*), thyroid peroxidase (*TPO*), thyroglobulin (*TG*), dehalogenase 1 (*DEHAL1*) and solute carrier families: 26 (*SLC26A4* or *PDS*) and 5 (*SLC5A5* or *NIS*) [1,4]. The synthesis of thyroid hormones (T_4_ and T_3_) is affected in 20% of all cases involving inborn genetic errors in the enzymatic cascade, which is defined as thyroid dyshormonogenesis [1]. Most often, these defects appear to be transmitted in an autosomal recessive manner [6], but autosomal dominant inheritance has also been reported [7].

Splicing is dependent on the exact identification of exons, which are perfectly recognized within pre-mRNAs. The presence of 5ʹ and 3ʹ splice sites and the branch points may not be sufficient to define intron-exon boundaries. The exonic elements are represented by exonic splicing enhancers (ESEs), where SR proteins bind and play a pivotal role in spliceosome assembly. Sequences that act as exonic splicing silencers (ESSs) bind to negative regulators, which belong to the heterogeneous nuclear ribonucleoprotein family. Both ESEs and ESSs appear to play an instrumental role in the regulation of alternative splicing events, other than the sequences that may play a pertinent role in the definition of constitutive exons [8].

Furthermore, microRNAs (miRs), which are 22‒23 nucleotides in length, bind to the 3ʹ-untranslated region (3ʹ-UTR) and regulate mRNAs post-transcriptionally, either by facilitating mRNA degradation or by inhibiting mRNA transcription [9]. More than 30% of genes encoding for proteins are regulated by miRs [10]. Any genetic variation in the 3'-UTR may interfere with miR binding to its target, thereby influencing the expression of the targeted gene [11].

In our previous study, we investigated the effects of *TSHR*, *TPO*, and *TG* genetic variants in CH and identified 22 variants [12]. Three of these 22 variants (p.S398T in *TPO* and p.G653D and p.R1999W in *TG*) were predicted to be deleterious [12]. In this study, we aimed to perform an *in silico* analysis to achieve a better understanding of polymorphic variants in the 3'-UTR of the *TSHR* gene, which interferes with the binding affinities of miRs that might inhibit gene expression. Further, we analyzed mutant protein structure-function relationships through molecular modeling and interaction studies for the p.S398T mutation of TPO. In addition, molecular modeling, mutation analyses, and thermodynamic energy calculations for variants of p.G653D and p.R1999W in the *TG* gene were also performed.

## Methods

As part of a newborn screening (NBS) program for CH, we analyzed 49,432 newborns, of whom 1,099 were screened with negative findings, along with 45 confirmed cases of CH. The institutional ethical committee of Rainbow Children’s Hospital, located in Hyderabad, India, approved the study protocol (RCHBH/066/02-2018). Informed consent was obtained from the parents or guardians of all neonates [12].

### *In silico* analysis of SNP-miR interactions

Four web-based tools—PolymiRTS (http://compbio.uthsc.edu/miRSNP/) [13], miRDB (http://www.mirdb.org/cgi-bin/search.cgi) [14], TargetScan (http://www.targetscan.org) [15], and STarMir (http://sfold.wadsworth.org) [16]—were used to ascertain whether the identified single-nucleotide polymorphisms (SNPs) in the 3'-UTR region interfered with miR binding.

### Molecular modeling and protein-protein docking of the TPO protein

To further analyze the predicted role of SNPs in the protein interactions, molecular modeling and interaction studies were carried out. The Uniprot IDs of TPO and DUOX1 are P07202 and Q9NRD9, respectively. As the structures of TPO and DUOX1 are not crystallized, model prediction for the functional domains of the proteins was carried using the I-Tasser server [17]. The TPO domain (residues 167‒734) of human TPO was modeled, as was the similarly interacting protein of the human dual peroxidase domain of DUOX. The 398 Ser-Thr mutant of TPO was modeled and energy-minimized using Chimera [18], and energy minimization was carried out using the steepest descent method. The modeled structures were docked using the ClusPro server [19] and the probable interactions were predicted using the PIC webserver [20]. The protein-protein interaction energies were calculated using the PRODIGY server [21].

### Molecular modeling of the wild-type and mutant TG protein

To understand the effect of SNPs such as p.G653D and p.R1999W on the TG protein, we performed molecular modeling, mutation analyses, and thermodynamic energy calculations using the SAAMBE-3D server [22]. The crystal structure of human TG was taken from the protein database (PDB ID: 6SCJ). The crystal structure was solved using electron microscopy with a resolution of 3.60 Å [23]. Mutations such as p.G653D and p.R1999W were inserted *in silico* into the wild-type TG protein using the ‘Mutagenesis’ wizard of the ‘PyMol’ software [24]. These modeled structures of mutant TG proteins were further used to understand the effects of the mutations on protein structural stability and its intramolecular interactions. The effect of mutations such as p.G653D and p.R1999W on TG protein stability were checked using the SAAMBE-3D server [22], which predicts the effects of mutations on protein stability. In addition, we used the DynaMut server [25] to understand the effects of mutations on various types of interactions, such as van der Waals, weak polar van der Waals, polar proximal, amide-amide interactions, and so on.

## Results and Discussion

In our previous studies, we established the reference intervals for thyroid-stimulating hormone (TSH) through NBS data, and the specific genotype-phenotype correlations were exhibited in confirmed CH cases with *TSHR*, *TPO*, and *TG* gene variants [12]. Previously, we published an *in silico* analysis on p.D727E in the *TSHR* gene, which might control the signal transduction (cAMP-mediated) pathway, consequently contributing to the pathophysiology of CH [26]. In this study, we specifically focused on an *in silico* analysis of three variants: p.S398T in TPO, and p.G653D and p.R1999W in TG.

In a study we reported earlier, we identified eight intronic variants (g. IVS 01+63 G>C, g.IVS 06-69 C>T, g.IVS 06+13 A>G, g.IVS 09+58 T>G in the *TSHR* gene, g.IVS 11+20 G>A, g.IVS 13+128 C>T, g.IVS 14‒37 G>A, g.IVS 14‒19 G>C in the *TPO* gene) [12]. The interpretation of the intronic variants is that g.IVS 01+63 G>C, g.IVS 06-69 C>T, g.IVS 09+58 T>C are involved in the creation of an intronic ESE site and g.IVS 06+13 A>G is an alteration of an intronic ESS site. The intronic variant g.IVS 11+20 G>A predicted a signal wherein an ESS site is broken and a new ESS site is created; this is interpreted as involving an alteration of an intronic ESS site and creation of an intronic ESE site. These results may not have an impact on splicing; no significant splicing motif alterations were detected for the remaining SNPs, which probably have no impact on the splicing mechanism (http://www.umd.be/HSF3/HSF.shtml).

### *In silico* analysis of SNP-miR interaction

The four SNPs in the 3'-UTR of the *TSHR* gene (rs2268477, rs373305430, rs7144481, and rs17630128) were predicted to alter the binding of 10 common miRs as per the data obtained from four different databases (PolymiRTS, miRDB, TargetScan, and STarMir). As shown in Table 1, the presence of the rs2268477 polymorphic variant destroys the binding site for hsa-miR-154-5p. The rs373305430, rs7144481, and rs17630128 polymorphic variants alter the binding affinities of hsa-miR-1237-5p, hsa-miR-4488, hsa-miR-4697-5p, hsa-miR-6846-5p, hsa-miR-6848-5p, hsa-miR-376a-2-5p, hsa-miR-3935, hsa-miR-4280, and hsa-miR-6858-3p. The 3'-UTR SNPs-miR interaction hybrid diagrams are shown in Supplementary Fig. 1.

The rs2268477, rs373305430, rs7144481, and rs17630128 SNPs were localized in the 3'-UTR region of the *TSHR* gene and hence associated with binding of the 10 different miRs. These SNPs in the miR target site on the 3'-UTR may affect the binding efficacy of miR. SNPs may alter target gene expression to affect post-transcriptional processing and polyadenylation, and they may even contribute to CH. Among the risk variants, rs2268477 was found to destroy the binding site of hsa-miR-154-5p. In papillary thyroid carcinoma, hsa-miR-154 was reported to be downregulated [27], which substantiates the role of miR-154 in thyroid development. The rs7144481 variant was found to alter binding affinities of hsa-miR-376a-2-5p, hsa-miR-3935 and miR-4280. Long non-coding RNA LINC00488, which is reported to be highly expressed in thyroid cancer cell lines, directly binds to miR-376a and downregulates its expression [28]. The rs17630128 variant was found to alter the binding affinity of hsa-miR-6858-3p.

The binding of TSH to TSHR induces angiogenesis by modulating vascular endothelial growth factor expression through cAMP‒mammalian target of rapamycin signaling [29]. In endurance athletes, the frequency of rs7144481 C-allele (wild-type allele) was reported to be higher than in controls, contributing to a high metabolic rate and better aerobic performance [30]. Through the regulation of gene expression, the rs7144481 SNP may decrease angiogenesis and/or the metabolic rate. Campo and his group found that rare allele carriers of rs7144481 in *TSHR* were at an increased risk of well-differentiated thyroid cancer [31].

### *In silico* predictions of function of the c. 1284 G>C (p.S398T) mutation in the TPO protein

**Fig. 1.**
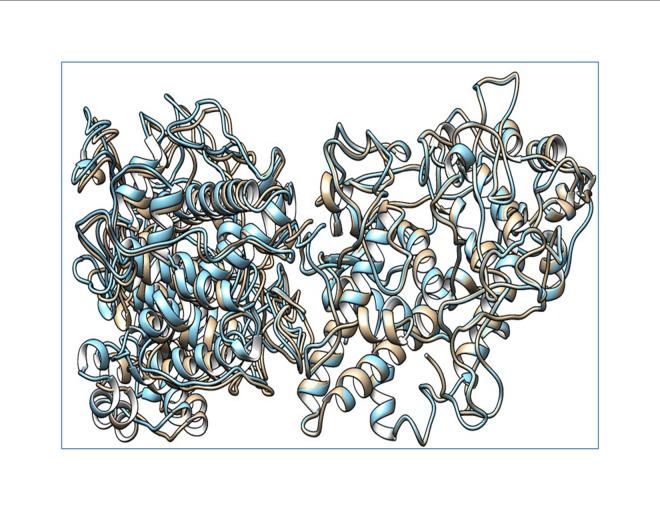
Alignment showing the wild-type thyroid peroxidase (TPO) and mutant TPO with dual oxidase. The wild-type complex is shown in blue and the mutant complex is shown as mutant TPO. Deviation in the complex is shown by the non-alignment of the structures.

The docked structures of the wild-type and mutant structures with DUOX were saved for further structure-based analysis. The predicted wild-type and mutant structures were aligned using chimera to analyze the structural variation caused by the mutation. Various interactions between the wild-type and the mutant protein are tabulated and provided in Supplementary Table 1. The binding energies and dissociation constants for the wild-type and mutant complexes were also analyzed to reveal the functional alterations induced by the SNPs (Fig. 1). The TPO structures show a fluctuation in the root-mean-square deviation (RMSD) of 0.152 Å and the interacting DUOX shows an RMSD of 1.091 Å. This shows that the SNP induces more structural variations in the binding protein DUOX than in TPO, which harbors the SNP. These structural variations induced in the binding complex alter the binding pattern which causes major variations in functional aspects of *TPO*. The mutant induces novel non-bonded hydrophobic interactions by the Ala263 residue, while there is a steep fall in the numbers of hydrophobic interactions by Trp200 and Leu267. A similar fall in the numbers of hydrogen-bonds involving main chain–main chain, main chain–side chain, and side chain–side chain patterns are observed. A steep fall in the number of ionic interactions by Arg198 with DUOX is observed in the mutant protein complex, whereas the aromatic–aromatic interactions and cation–pi interactions are retained. The complete list of the molecular interactions between the TPO wild-type and mutant proteins and the DUOX protein are given in Supplementary Table 1. These altered interactions further decrease the binding energies. The wild-type complex showed a ΔG of ‒15 kcal/mol and a dissociation constant (K_d_) of 7.2E^-12^ M, and the mutant showed a ΔG of ‒13.8 kcal/mol and a K_d_ of 7.0E^-11^ M. These binding energies and dissociation constants further show that the mutant complex is less stable and dissociates more easily than the wild-type protein. The *in silico* analysis of p.S398T revealed that the mutant protein complex is less stable and it may be deleterious.

TPO and TG play a pivotal role in the biosynthesis of thyroid hormones by supplying hydrogen peroxide (H_2_O_2_) and by serving as an iodine acceptor [32]. TPO protein activity hinges on the proper folding and insertion of the membrane, as well as a complete catalytic site with the heme-binding region, which is encoded by exons 8, 9, and 10 [33]. *TPO* gene-inactivating mutations lead to iodide organification defect (IOD), which is either partial (PIOD) or total (TIOD) depending on the mutation type and position. IOD is diagnosed by a positive perchlorate discharge test (PDT) [34]. Turkkahraman et al. [35] performed PDT after the fourth week of the LT_4_ period. They found that one patient (variant-p.S398T) had PIOD (24.4%) with positive PDT (normal range, <10%). They suggested that PIOD should be considered in infants with permanent subclinical hypothyroidism [35].

*TPO* c.1284G>C (p.S398T) is located in the eighth exon of *TPO*, which is part of the heme-binding catalytic site of *TPO*. Hence, the decreased interaction stability results in impaired binding to heme and affects its interaction with iodide and TG [36]. Guriaet al. demonstrated that p.A373S, p.S398T, and p.T725P had damaging effects on *TPO* mRNA expression and protein activity [37]. Furthermore, they measured wild-type and mutant enzyme activity using an iodide and guaiacol assay and confirmed that the p.A373S and p.T725P mutants were more damaging than the p.S398T mutant [37]. Begum et al. performed an analysis of quantum mechanics/molecular mechanics and molecular dynamics on p.A373S, p.S398T, and p.T725P. This molecular docking study showed that the full-length TPO mutant p.S398T structure interacted with all the crucial amino acids in the catalytic site of the TPO protein. Begum et al. [38] concluded that the mutant variants p.A373S, p.S398T, and p.T725P were involved in Bangladeshi patients with thyroid dyshormonogenesis, and their molecular docking–based study showed that the three mutant variants had damaging effects on the activity of the TPO protein. In our study, the protein structure of TPO, along with that of *DUOX1*, was crystallized, and the mutant protein complex was predicted to be less stable, which may have an effect on the function of the protein. The mutant protein p.S398T was analyzed for binding energies and dissociation constants for wild-type versus mutant complexes, with results of ΔG ‒15 vs. ‒13.8 kcal/mol and K_d_ 7.2E^-12^ vs. 7.0E^-11^ M, respectively. The RMSD of the TPO protein structure is high (1.091 Å) when it interacted with the DUOX protein, which illuminates the fact that the variant induced more structural variations in the binding protein DUOX than in TPO, which harbored the variant.

### *In silico* predictions of function of c.1999 G>A (p.G653D) and c.6036 C>T (p.R1999W) in the TG protein

No three-dimensional structures are available for any TG regions [39]. Three-dimensional structural folding of a long-chain amino acid sequence was seen as a complex problem in the past [40]. Due to high molecular weight of TG and the lack of its crystal structure, *in silico* studies on TG have not been conducted to date. The recent elucidation of its crystal structure (PDB ID: 6SCJ) [23] facilitated the *in silico* exploration of *TG* variants. The predicted wild-type and mutant (p.G653D and p.R1999W) structures of TG proteins were aligned using the PyMol software [24] to analyze the effects of structural variation caused by the mutation in the surrounding residues within 4 Å.

#### Analysis of the effect of the p.G653D mutation

**Fig. 2.**
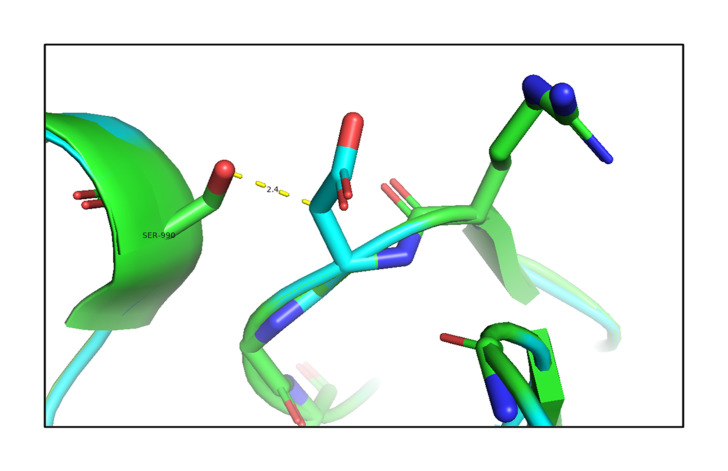
An *in silico* analysis shows overlapping images of the wild-type (green) and mutant G653D (cyan) thyroglobulin protein. Here, the mutant residue Asp653 is shown in a stick model with hydrogen-bonding interactions with the Ser990 residue.

The analysis of the structure of the p.G653D mutant of TG showed that the mutation of Gly653 to Asp653 formed hydrogen-bonding interactions with the surrounding residue Ser990 (2.4 Å) as shown in Fig. 2, resulting in altered TG protein structure (Supplementary Video 1). The SAAMBE-3D prediction showed that the mutation destabilized the TG protein, with a ΔG value of 0.24 kcal/mol.

**Fig. 3.**
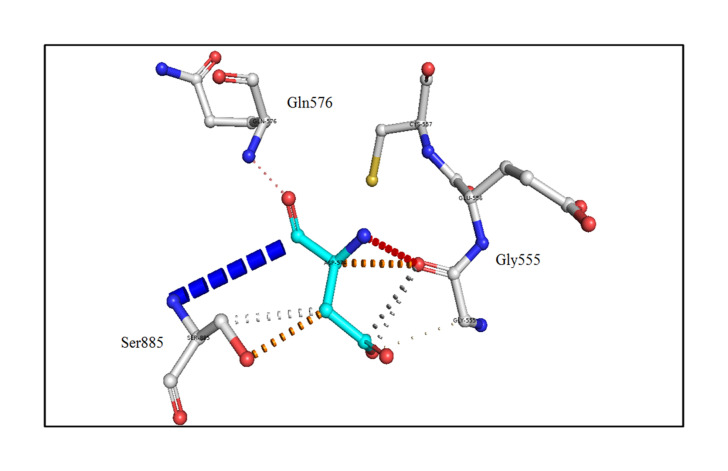
An *in silico* analysis shows the mutant residue Asp653 colored in cyan, which is also represented as sticks alongside with the surrounding residues colored in white, which are involved in any type of interactions. The mutant thyroglobulin protein shows the weak polar van der Waals clashes with the residues Gly555 and Ser885, polar proximal interactions with Gln576, and amide-amide interactions with Ser885.

Next, the effect of the mutation on the adjacent residues was investigated using the DynaMut server [25]. The mutant Asp653 formed a weak polar van der Waals bond with the residues Gly555 and Ser885, polar proximal interactions with Gln576, and amide-amide interactions with Ser885, as shown in Fig. 3.

#### Analysis of the effect of the p.R1999W mutation

**Fig. 4.**
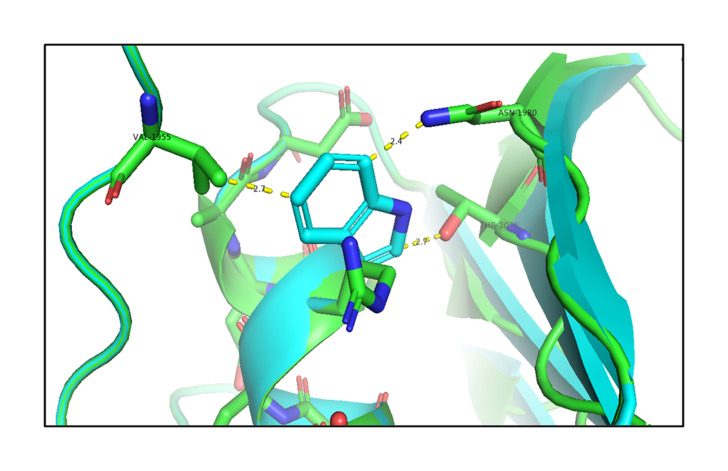
An *in silico* analysis shows the overlapped images of wild-type (green) and mutant R1999W (cyan) thyroglobulin protein. The wild-type Arg1999 is shown in green and the mutant Trp1999 is shown in cyan. The mutant Trp1999 forms bonding interactions with Val1955, Asn1980, and Thr2026.

Next, the analysis of the mutant (p.R1999W) TG structures showed that the mutation of Arg1999 to Trp1999 formed hydrogen-bonding interactions with the surrounding residues, such as Val1955 (2.7 Å), Asn1980 (2.4 Å), and Thr2026 (2.7 Å) shown in Fig. 4 (Supplementary Video 2). This shows that the mutation of p.R1999W resulted in bonding interactions with the surrounding residues, which may affect the structure-function relationship of the TG protein. The effect of the p.R1999W mutation on protein stability was further assessed using the SAAMBE-3D server. The SAAMBE-3D prediction showed that the mutation destabilized the *TG* protein and the ΔG value was 0.79 kcal/mol.

**Fig. 5.**
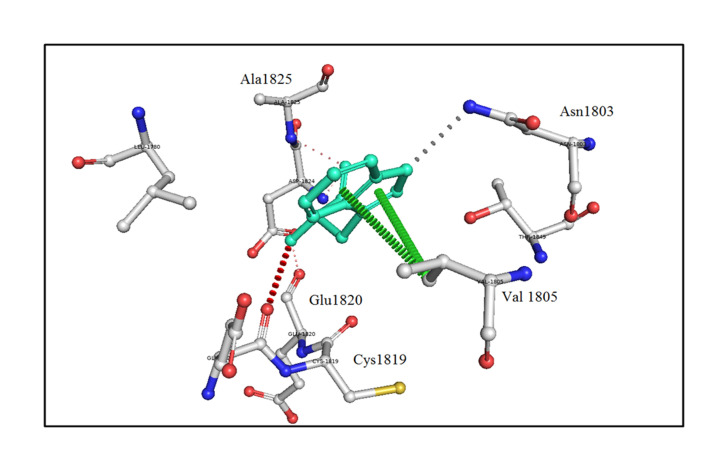
An *in silico* analysis shows the mutant p.R1999W residue colored in light-green and also represented as sticks alongside with the surrounding residues colored in white, which are involved in any type of interactions. Here, Trp1999 shows van der Waals clashes with Glu1820 and Thr1845, hydrogen-bond and van der Waals clashes with Glu1818, hydrophobic proximal clashes with Val1805 and Leu1780, hydrogen-bond proximal interactions with Ala1825, van der Waals clashes with Asn1803, and carbon-pi interactions with Val1805.

The analysis of bonding and non-bonding interactions shows that the p.R1999W mutation results in non-bonding interactions with the residues; for instance, Trp1999 shows van der Waals clashes with Glu1820 and Thr1845, hydrogen-bond and van der Waals clashes with Glu1818, hydrophobic proximal clashes with Val1805 and Leu1780, hydrogen-bond proximal interactions with Ala1825, van der Waals clashes with Asn1803, and carbon-pi interactions with Val1805 (Fig. 5).

These altered interactions due to the p.G653D and p.R1999W mutations decrease the binding energies; here, the mutation of p.R1999W shows a more destabilizing effect on the TG protein due to more hydrogen-bonding interactions (Fig. 4), van der Waals and carbon-pi interactions (Fig. 5), and a higher binding energy (ΔG = 0.79 kcal/mol) compared to the p.G653D mutation (ΔG = 0.24 kcal/mol). These structural and energetic analyses show that the mutants are less stable and affect the structure-functional relationship of the human TG protein.

Aljouie et al. studied synonymous and nonsynonymous variants such as rs76672487 in the *ABCC2* gene and rs2069548 (p.G653D) in the *TG* gene. According to the Human Protein Atlas, these two genes are cancer-related genes. The variant p.G653D was ranked fourth of the selected variants using the chi-square test from the top SNPs in the glioblastoma multiforme +1000 genome database. The nearby tissue was enriched in this rare variant. This variant affects cancer susceptibility by suppressing mRNA expression (transcription and translation) [41]. Autoimmune thyroid diseases, including hyperthyroidism/Graves’ disease and autoimmune hypothyroidism/Hashimoto’s thyroiditis, are complex diseases caused by a malfunction in immune tolerance to self-thyroid antigens, such as TSHR, TPO, and TG [42]. Pyunet al. [43] identified epistasis between two polymorphic variants of the *HSD17B4* and *TG* genes. One variant, p.R1999W (c.5995 C>T), in the *TG* gene was tested for epistasis with the *HSD17B4* variant p.T687I (c.2060 C>T). The combined effect of these variants was significantly associated with premature ovarian failure, although these variants alone showed no significant association. Ban et al. [[44] performed case-control association studies for 14 discovered *TG* variants in 285 autoimmune thyroid disease patients and 150 controls. One variant cluster (p.S753A & p.P797P in exon 10 and p.M1027V in exon 12) [44] and the exon 33 variant p.R1999W showed significant associations with patients suffering from autoimmune thyroid disease [44,45] in the United States [44], but were not associated with patients in the United Kingdom suffering from the same disease [42]. The combination of these variants conferred susceptibility to autoimmune thyroid disease. Ban et al. [44] analyzed gene-gene interactions between p.R1999W in the *TG* gene and *HLA-DR3*. The variant (p.R1999W) showed promising results for interaction with *HLA-DR3* in conferring susceptibility to Graves’ disease (odds ratio, 6.1) [44].

Although there are no reports depicting the direct association of p.G653D and p.R1999W variants of the TG protein with CH, several recent studies have illuminated the role of TG variants in the etiology of CH. These studies illustrate TG as one of the primary candidate genes to be evaluated in CH patients [46-49]. In our study, we investigated the effect of mutations such as p.G653D and p.R1999W, which were analyzed using computational structural biology methods, including mutant model building for the TG protein and structural analysis of interaction networks such as hydrogen-bonding. A further analysis of binding energies using the SAAMBE-3D server demonstrated that the p.G653D and p.R1999W mutations showed a protein destabilizing effect, which revealed that these SNPs may be deleterious and affect the TG protein structure-function relationship.

In conclusion, *in silico* studies revealed that SNPs in the 3'-UTR region altered the binding affinity of various miRs, thus influencing the expression of thyroid-associated genes. In this study, analyses of the computational protein-protein interactions and the binding energies of the p.S398T mutation in the *TPO* gene showed that the mutant protein complex was less stable than the wild-type complex, implying that this SNP may be deleterious. The altered variants p.G653D and p.R199W decreased the binding energies and contributed to a destabilizing effect on the TG protein.

## Figures and Tables

**Table 1.** *In silico* studies revealing *TSHR* SNP-miRNA interactions

Gene	SNP	miRNA	Seed match	Wild	ΔG (kcal/mol)	Mutant	ΔG (kcal/mol)
*TSHR*	rs2268477	hsa-miR-154-5p	7-mer	uauUAA**C**CUAa	‒22.0	Disruption of binding site	
	rs373305430	hsa-miR-1237-5p	6-mer	auu**G**CCCCCa	‒23.4	auu**U**CCCCCa	‒17.4
		hsa-miR-4488	6-mer	auu**G**CCCCCa	‒25.8	auu**U**CCCCCa	‒15.9
		hsa-miR-4697-5p	6-mer	auu**G**CCCCCa	‒29.8	auu**U**CCCCCa	‒22.8
		hsa-miR-6846-5p	6-mer	auu**G**CCCCCa	‒29.6	auu**U**CCCCCa	‒17.3
		hsa-miR-6848-5p	6-mer	auu**G**CCCCCa	‒26.3	auu**U**CCCCCaa	‒22.0
	rs7144481	hsa-miR-376a-2-5p	8-mer	auAAUCUA**C**A	‒17.5	auAAUCUA**U**A	‒16.1
		hsa-miR-3935	7-mer	auaAUCUA**C**Ac	‒18.7	auaAUCUA**U**Ac	‒14.0
		hsa-miR-4280	7-mer	aauCUA**C**ACUa	‒19.6	aauCUA**U**ACUa	‒17.7
	rs17630128	hsa-miR-6858-3p	7-mer	cacG**U**UGGCUc	‒20.2	cacG**C**UGGCUc	‒22.6

In the table given above, capital letters of the miR site indicates the miRNA binding site of the 3ʹ-UTR. Bold letters indicate SNPs identified in our study. *TSHR*, thyroid stimulating hormone receptor; SNP, single-nucleotide polymorphism; miRNA, microRNA; ΔG, binding energy; mer, nucleotide pairing.

## Supplementary Materials

Supplemental Table. 1. Molecular modeling and protein-protein docking was shown on wild TPO and mutant TPO with DUOX.

Supplemental Fig. 1. Schematic representation of the miRNA binding site on the 3'-UTR variants found in the *TSHR* gene. Hybrid diagram for a seed site (miRNA seed region in red) and hybrid diagram for a seedless (non-canonical) site. (A) rs2268477/c.172 C>A (hsa-miR-154-5p), which can destroy the binding site. (B) rs373305430/c.182 G>T (hsa-miR-1237-5p; hsa-miR-4488; hsa-miR-4697-5p; hsa-miR-6846-5p; hsa-miR-6848-5p). (C) rs7144481/c.245 C>T (hsa-miR-376a-2-5p; hsa-miR-3935; hsa-miR-4280). (D) rs17630128/c.431 T>C (hsa-miR-6858-3p).

Supplemental Video 1. The analysis of effect of mutant TG protein (p.G653D) showed that the mutation of Gly653 to Asp653 forms the H-bonding interactions with the surrounding residue Ser990.

Supplemental Video 2. The analysis of effect of mutant TG protein (p.R1999W) showed that the mutation of Arg1999 to Trp1999 forms the H-bonding interactions with the surrounding residues such as Val1955, Asn1980, and Thr2026.
