# Supplementary material for: Molecular insights into the role of genetic determinants of congenital hypothyroidism
Source: Genomics Inform. 2021 Sep 30;19(3):e29. doi: 10.5808/gi.21034 (PMC8510868; doi:10.5808/gi.21034)
Supplement: Supplemental Table. 1. — Molecular modeling and protein-protein docking was shown on wild TPO and mutant TPO with DUOX. [file gi-21034suppl1.pdf]

Wild TPO - Duox Interactions

Protein-Protein Hydrophobic Interactions

[help]

Rasmol Jmol

Wild_Dock.pdb

Hydrophobic Interactions within 5 Angstroms

Position	Residue	Chain	Position	Residue	Chain
200	TRP	A	2	TRP	B
200	TRP	A	4	VAL	B
200	TRP	A	559	VAL	B
202	ALA	A	7	PHE	B
205	ALA	A	2	TRP	B
266	ALA	A	15	MET	B
266	ALA	A	287	PRO	B
266	ALA	A	288	PHE	B
267	LEU	A	15	MET	B
267	LEU	A	288	PHE	B

NO PROTEIN-PROTEIN DISULPHIDE BRIDGES FOUND

Protein-Protein Main Chain-Main Chain Hydrogen Bonds

[help]

Rasmol Jmol

[View the original hbond output]

Wild_Dock.pdb

DONOR				ACCEPTOR			
POS	CHAIN	RES	ATOM	POS	CHAIN	RES	ATOM
558	B	VAL	N	197	A	THR	O
559	B	VAL	N	197	A	THR	O

Dd-a = Distance Between Donor and Acceptor

Dh-a = Distance Between Hydrogen and Acceptor

A(d-H-N) = Angle Between Donor-H-N

A(a-O=C) = Angle Between Acceptor-O=C

MO = Multiple Occupancy

Note that angles that are undefined are written as 999.99

Protein-Protein Main Chain-Side Chain Hydrogen Bonds

[help]

Rasmol Jmol
[View the original hbond output]
Wild_Dock.pdb

DONOR				ACCEPTOR			
POS	CHAIN	RES	ATOM	POS	CHAIN	RES	ATOM
197	A	THR	OG1	557	B	SER	O
198	A	ARG	NH1	244	B	ASP	O
198	A	ARG	NH1	244	B	ASP	O
198	A	ARG	NH2	244	B	ASP	O
198	A	ARG	NH2	244	B	ASP	O
202	A	ALA	N	248	B	GLU	OE2
203	A	GLY	N	252	B	GLN	OE1
211	A	ARG	NH2	568	B	PHE	O
211	A	ARG	NH2	568	B	PHE	O
259	A	ARG	N	11	B	TYR	OH
6	B	ARG	NH1	538	A	CYS	O
6	B	ARG	NH1	538	A	CYS	O
6	B	ARG	NH2	538	A	CYS	O
6	B	ARG	NH2	538	A	CYS	O
6	B	ARG	NH2	539	A	ASP	O
6	B	ARG	NH2	539	A	ASP	O
6	B	ARG	NE	541	A	THR	O
6	B	ARG	NH1	541	A	THR	O
6	B	ARG	NH1	541	A	THR	O
6	B	ARG	NH2	541	A	THR	O
6	B	ARG	NH2	541	A	THR	O
11	B	TYR	OH	255	A	ARG	O
18	B	ARG	NH2	563	A	CYS	O
18	B	ARG	NH2	563	A	CYS	O
248	B	GLU	OE2	200	A	TRP	O
248	B	GLU	OE2	200	A	TRP	O
561	B	ASP	OD1	197	A	THR	O
561	B	ASP	OD1	197	A	THR	O

Dd-a = Distance Between Donor and Acceptor
Dh-a = Distance Between Hydrogen and Acceptor
A(d-H-N) = Angle Between Donor-H-N
A(a-O=C) = Angle Between Acceptor-O=C
MO = Multiple Occupancy
Note that angles that are undefined are written as 999.99

Protein-Protein Side Chain-Side Chain Hydrogen Bonds
[help]

Rasmol Jmol

[View the original hbond output]

Wild_Dock.pdb

DONOR				ACCEPTOR			
POS	CHAIN	RES	ATOM	POS	CHAIN	RES	ATOM
198	A	ARG	NE	244	B	ASP	OD2
198	A	ARG	NH1	246	B	GLU	OE1
198	A	ARG	NH1	246	B	GLU	OE1
198	A	ARG	NH1	246	B	GLU	OE2
198	A	ARG	NH1	246	B	GLU	OE2
198	A	ARG	NH2	246	B	GLU	OE1
198	A	ARG	NH2	246	B	GLU	OE1
199	A	HIS	ND1	246	B	GLU	OE2
211	A	ARG	NH1	564	B	GLU	OE1
211	A	ARG	NH1	564	B	GLU	OE1
211	A	ARG	NH1	564	B	GLU	OE2
211	A	ARG	NH1	564	B	GLU	OE2
211	A	ARG	NH2	564	B	GLU	OE2
211	A	ARG	NH2	564	B	GLU	OE2
258	A	ASN	ND2	1	B	SER	OG
258	A	ASN	ND2	1	B	SER	OG
259	A	ARG	NH1	17	B	HIS	NE2
259	A	ARG	NH1	17	B	HIS	NE2
382	A	ARG	NH2	158	B	HIS	NE2
382	A	ARG	NH2	158	B	HIS	NE2
1	B	SER	OG	258	A	ASN	ND2
10	B	TRP	NE1	539	A	ASP	OD2
18	B	ARG	NE	564	A	GLU	OE2
18	B	ARG	NH2	564	A	GLU	OE2
18	B	ARG	NH2	564	A	GLU	OE2
246	B	GLU	OE2	199	A	HIS	ND1
246	B	GLU	OE2	199	A	HIS	ND1

Dd-a = Distance Between Donor and Acceptor

Dh-a = Distance Between Hydrogen and Acceptor

A(d-H-N) = Angle Between Donor-H-N

A(a-O=C) = Angle Between Acceptor-O=C

MO = Multiple Occupancy

Note that angles that are undefined are written as 999.99

Protein-Protein Ionic Interactions

[help]

Rasmol Jmol

Wild_Dock.pdb

Ionic Interactions within 6 Angstroms

Position	Residue	Chain	Position	Residue	Chain
198	ARG	A	244	ASP	B
198	ARG	A	246	GLU	B
198	ARG	A	561	ASP	B
199	HIS	A	246	GLU	B
199	HIS	A	248	GLU	B
204	ARG	A	162	ASP	B
211	ARG	A	564	GLU	B
378	ASP	A	158	HIS	B
539	ASP	A	17	HIS	B
539	ASP	A	6	ARG	B
564	GLU	A	18	ARG	B

Protein-Protein Aromatic-Aromatic Interactions
[help]
Rasmol Jmol
Wild_Dock.pdb

Aromatic-Aromatic Interactions within 4.5 and 7 Angstroms

Residue	Position	Chain	Residue	Position	Chain	D(centroid-centroid)	
200	TRP	A	2	TRP	B	6.01	79.01

NO PROTEIN-PROTEIN AROMATIC-SULPHUR INTERACTIONS FOUND

Protein-Protein Cation-Pi Interactions
[help]
Rasmol Jmol
Wild_Dock.pdb

Cation-Pi Interactions within 6 Angstroms

Position	Residue	Chain	Position	Residue	Chain	D(cation-Pi)	Angle
2	TRP	B	255	ARG	A	5.87	57.47

		PARAMETERS		
MO	Dd-a	Dh-a	A(d-H-N)	A(a-O=C)
-	3.3	4.02	37.85	150.79
-	2.88	1.91	158.04	149.16

PARAMETERS

MO		Dd-a	Dh-a	A(d-H-N)	A(a-O=C)
-		2.75	9.99	999.99	164.65
	1	3.39	4.18	36.47	115.04
	2	3.39	3.12	96.43	115.04
	1	2.86	3.37	52.32	119.38
	2	2.86	2.54	97.35	119.38
-		3.05	2.08	163.54	115.19
-		3.1	2.84	95.63	101.33
	1	2.63	2.02	114.29	126.11
	2	2.63	2.55	82.88	126.11
-		3.24	3.17	84.87	999.99
	1	3.4	4.38	18.64	175.09
	2	3.4	2.74	123.47	175.09
	1	2.72	3.4	42.33	141.84
	2	2.72	1.79	149.36	141.84
	1	2.82	2.06	127.6	89.62
	2	2.82	2.96	72.42	89.62
-		3.33	4.03	39.23	151.52
	1	2.73	3	65.4	158.93
	2	2.73	2.99	65.81	158.93
	1	3.31	3.9	49.04	153.99
	2	3.31	3.55	68.06	153.99
-		2.93	9.99	999.99	132.21
	1	2.89	3.13	67.53	87.27
	2	2.89	2.06	136.16	87.27
	1	3.24	4.01	37.79	129.91
	2	3.24	2.64	113.48	129.91
	1	3.15	2.13	157.13	138.91
	2	3.15	3.56	59.36	138.91

PARAMETERS

MO		Dd-a	Dh-a	A(d-H-N)	A(a-O=C)
-		2.91	2.34	116.71	999.99
	1	2.82	3.73	25.78	999.99
	2	2.82	2.04	132.08	999.99
	1	2.76	3.22	55.2	999.99
	2	2.76	1.78	159.96	999.99
	1	2.74	3.61	28.78	999.99
	2	2.74	1.9	137.25	999.99
-		2.88	2	163.24	999.99
	1	2.8	3.41	46.95	999.99
	2	2.8	1.86	155.61	999.99
	1	2.96	3.91	22.42	999.99
	2	2.96	2.2	131.73	999.99
	1	2.71	3.54	32.19	999.99
	2	2.71	1.8	146.53	999.99
	1	2.75	3.38	45.73	999.99
	2	2.75	1.91	135.74	999.99
	1	2.86	2.99	73.13	999.99
	2	2.86	2.17	124.18	999.99
	1	2.84	3.32	54.54	999.99
	2	2.84	1.97	143.41	999.99
-		2.75	9.99	999.99	999.99
-		2.91	2.07	153.3	999.99
-		2.9	2.26	120.72	999.99
	1	2.7	1.79	142.99	999.99
	2	2.7	3.52	31.62	999.99
	1	2.88	2.96	75.12	999.99
	2	2.88	2.9	78.17	999.99

Dihedral Angle

Mutant TPO - Duox Interactions

Protein-Protein Hydrophobic Interactions

[help]

Rasmol Jmol

Mutant_Dock.pdb

Hydrophobic Interactions within 5 Angstroms

Position	Residue	Chain	Position	Residue	Chain
200	TRP	A	2	TRP	B
200	TRP	A	4	VAL	B
202	ALA	A	7	PHE	B
205	ALA	A	2	TRP	B
263	ALA	A	15	MET	B
266	ALA	A	14	LEU	B
266	ALA	A	15	MET	B
267	LEU	A	15	MET	B

NO PROTEIN-PROTEIN DISULPHIDE BRIDGES FOUND

Protein-Protein Main Chain-Main Chain Hydrogen Bonds

[help]

Rasmol Jmol

[View the original hbond output]

Mutant_Dock.pdb

DONOR				ACCEPTOR			
POS	CHAIN	RES	ATOM	POS	CHAIN	RES	ATOM
559	B	VAL	N	197	A	THR	O

Dd-a = Distance Between Donor and Acceptor

Dh-a = Distance Between Hydrogen and Acceptor

A(d-H-N) = Angle Between Donor-H-N

A(a-O=C) = Angle Between Acceptor-O=C

MO = Multiple Occupancy

Note that angles that are undefined are written as 999.99

Protein-Protein Main Chain-Side Chain Hydrogen Bonds

[help]

Rasmol Jmol

[View the original hbond output]

Mutant_Dock.pdb

		DONOR				ACCEPTOR	
POS	CHAIN	RES	ATOM	POS	CHAIN	RES	ATOM
197	A	THR	OG1	557	B	SER	O
198	A	ARG	NH1	244	B	ASP	O
198	A	ARG	NH1	244	B	ASP	O
199	A	HIS	NE2	246	B	GLU	O
202	A	ALA	N	252	B	GLN	NE2
203	A	GLY	N	252	B	GLN	OE1
211	A	ARG	NH2	568	B	PHE	O
211	A	ARG	NH2	568	B	PHE	O
1	B	SER	OG	258	A	ASN	O
6	B	ARG	NH2	538	A	CYS	O
6	B	ARG	NH2	538	A	CYS	O
6	B	ARG	NH1	541	A	THR	O
6	B	ARG	NH1	541	A	THR	O
6	B	ARG	NH2	541	A	THR	O
6	B	ARG	NH2	541	A	THR	O
11	B	TYR	OH	259	A	ARG	O
18	B	ARG	NH2	563	A	CYS	O
18	B	ARG	NH2	563	A	CYS	O
248	B	GLU	N	199	A	HIS	NE2
249	B	GLU	N	199	A	HIS	NE2
561	B	ASP	OD1	197	A	THR	O
561	B	ASP	OD1	197	A	THR	O

Dd-a = Distance Between Donor and Acceptor

Dh-a = Distance Between Hydrogen and Acceptor

A(d-H-N) = Angle Between Donor-H-N

A(a-O=C) = Angle Between Acceptor-O=C

MO = Multiple Occupancy

Note that angles that are undefined are written as 999.99

Protein-Protein Side Chain-Side Chain Hydrogen Bonds

[help]

Rasmol Jmol

[View the original hbond output]

Mutant_Dock.pdb

		DONOR				ACCEPTOR	
POS	CHAIN	RES	ATOM	POS	CHAIN	RES	ATOM
198	A	ARG	NH1	246	B	GLU	OE1
198	A	ARG	NH1	246	B	GLU	OE1
198	A	ARG	NH1	246	B	GLU	OE2

198	A	ARG	NH1	246	B	GLU	OE2
198	A	ARG	NH2	246	B	GLU	OE1
198	A	ARG	NH2	246	B	GLU	OE1
198	A	ARG	NH2	246	B	GLU	OE2
198	A	ARG	NH2	246	B	GLU	OE2
199	A	HIS	ND1	246	B	GLU	OE2
211	A	ARG	NH1	564	B	GLU	OE2
211	A	ARG	NH1	564	B	GLU	OE2
211	A	ARG	NH2	564	B	GLU	OE2
211	A	ARG	NH2	564	B	GLU	OE2
259	A	ARG	NH1	17	B	HIS	NE2
259	A	ARG	NH1	17	B	HIS	NE2
382	A	ARG	NH2	158	B	HIS	NE2
382	A	ARG	NH2	158	B	HIS	NE2
10	B	TRP	NE1	539	A	ASP	OD2
18	B	ARG	NE	564	A	GLU	OE2
18	B	ARG	NH2	564	A	GLU	OE2
18	B	ARG	NH2	564	A	GLU	OE2
246	B	GLU	OE2	199	A	HIS	ND1
246	B	GLU	OE2	199	A	HIS	ND1

Dd-a = Distance Between Donor and Acceptor
Dh-a = Distance Between Hydrogen and Acceptor
A(d-H-N) = Angle Between Donor-H-N
A(a-O=C) = Angle Between Acceptor-O=C
MO = Multiple Occupancy
Note that angles that are undefined are written as 999.99

Protein-Protein Ionic Interactions
[help]
Rasmol Jmol
Mutant_Dock.pdb

Ionic Interactions within 6 Angstroms

Position	Residue	Chain	Position	Residue	Chain
198	ARG	A	246	GLU	B
199	HIS	A	246	GLU	B
204	ARG	A	8	ASP	B
211	ARG	A	564	GLU	B
378	ASP	A	158	HIS	B
539	ASP	A	17	HIS	B
564	GLU	A	18	ARG	B

Protein-Protein Aromatic-Aromatic Interactions

[help]
Rasmol Jmol
Mutant_Dock.pdb

Aromatic-Aromatic Interactions within 4.5 and 7 Angstroms

Residue	Position	Chain	Residue	Position	Chain	D(centroid-centroid)	
200	TRP	A	2	TRP	B	6.85	94.96

NO PROTEIN-PROTEIN AROMATIC-SULPHUR INTERACTIONS FOUND

Protein-Protein Cation-Pi Interactions
[help]
Rasmol Jmol
Mutant_Dock.pdb

Cation-Pi Interactions within 6 Angstroms

Position	Residue	Chain	Position	Residue	Chain	D(cation-Pi)	Angle
2	TRP	B	255	ARG	A	5.77	40.8

PARAMETERS				
MO	Dd-a	Dh-a	A(d-H-N)	A(a-O=C)
-	2.76	1.8	153.86	171.52

		PARAMETERS		
MO	Dd-a	Dh-a	A(d-H-N)	A(a-O=C)
-	2.72	9.99	999.99	153.65
1	2.64	2.07	111.77	166.22
2	2.64	2.98	60.61	166.22
-	3.26	2.37	168.61	82.7
-	3.44	2.94	112.18	94.79
-	3.04	2.67	102.88	112.02
1	2.69	1.72	151.07	141.69
2	2.69	3.09	57.6	141.69
-	2.89	9.99	999.99	96.61
1	3.37	3.69	64.07	160.2
2	3.37	2.44	151.28	160.2
1	2.63	3.33	40.79	134.94
2	2.63	2.32	95.96	134.94
1	2.78	3.54	37.22	164.87
2	2.78	2.52	93.91	164.87
-	3.15	9.99	999.99	107.17
1	3.11	3.22	74.6	85.72
2	3.11	2.42	124.12	85.72
-	3.33	2.81	115.15	999.99
-	3.33	2.41	164.87	999.99
1	3.31	2.28	161.54	116.15
2	3.31	4.02	43.2	116.15

		PARAMETERS		
MO	Dd-a	Dh-a	A(d-H-N)	A(a-O=C)
1	3.38	4.32	22.66	999.99
2	3.38	2.64	129.12	999.99
1	2.74	3.51	36.15	999.99

	2	2.74	1.87	140.94	999.99
	1	2.84	3.59	38.24	999.99
	2	2.84	1.93	149.29	999.99
	1	3.11	4.06	22.25	999.99
	2	3.11	2.4	127.03	999.99
-		3.26	3.57	63.03	999.99
	1	2.72	3.64	24.86	999.99
	2	2.72	1.89	137.88	999.99
	1	2.72	3.62	26.84	999.99
	2	2.72	1.87	138.44	999.99
	1	2.86	2.78	83.64	999.99
	2	2.86	2.31	112.64	999.99
	1	2.91	3.39	54.59	999.99
	2	2.91	1.96	155.97	999.99
-		3.18	2.37	148.44	999.99
-		2.85	2.15	126.12	999.99
	1	2.71	1.79	144.99	999.99
	2	2.71	3.55	30.16	999.99
	1	3.26	3.12	87.68	999.99
	2	3.26	2.49	126.51	999.99

Dihedral Angle
