# Supplementary material for: Molecular insights into the role of genetic determinants of congenital hypothyroidism
Source: Genomics Inform. 2021 Sep 30;19(3):e29. doi: 10.5808/gi.21034 (PMC8510868; doi:10.5808/gi.21034)
Supplement: Supplemental Fig. 1. — Schematic representation of the miRNA binding site on the 3'-UTR variants found in the TSHR gene. Hybrid diagram for a seed site (miRNA seed region in red) and hybrid diagram for a seedless (non-canonical) site. (A) rs2268477/c.172 C>A (hsa-miR-154-5p), which can destroy the binding site. (B) rs373305430/c.182 G>T (hsa-miR-1237-5p; hsa-miR-4488; hsa-miR-4697-5p; hsa-miR-6846-5p; hsa-miR-6848-5p). (C) rs7144481/c.245 C>T (hsa-miR-376a-2-5p; hsa-miR-3935; hsa-miR-4280). (D) rs17630128/c.431 T>C (hsa-miR-6858-3p). [file gi-21034suppl2.pdf]

Supplementary Fig. 1.

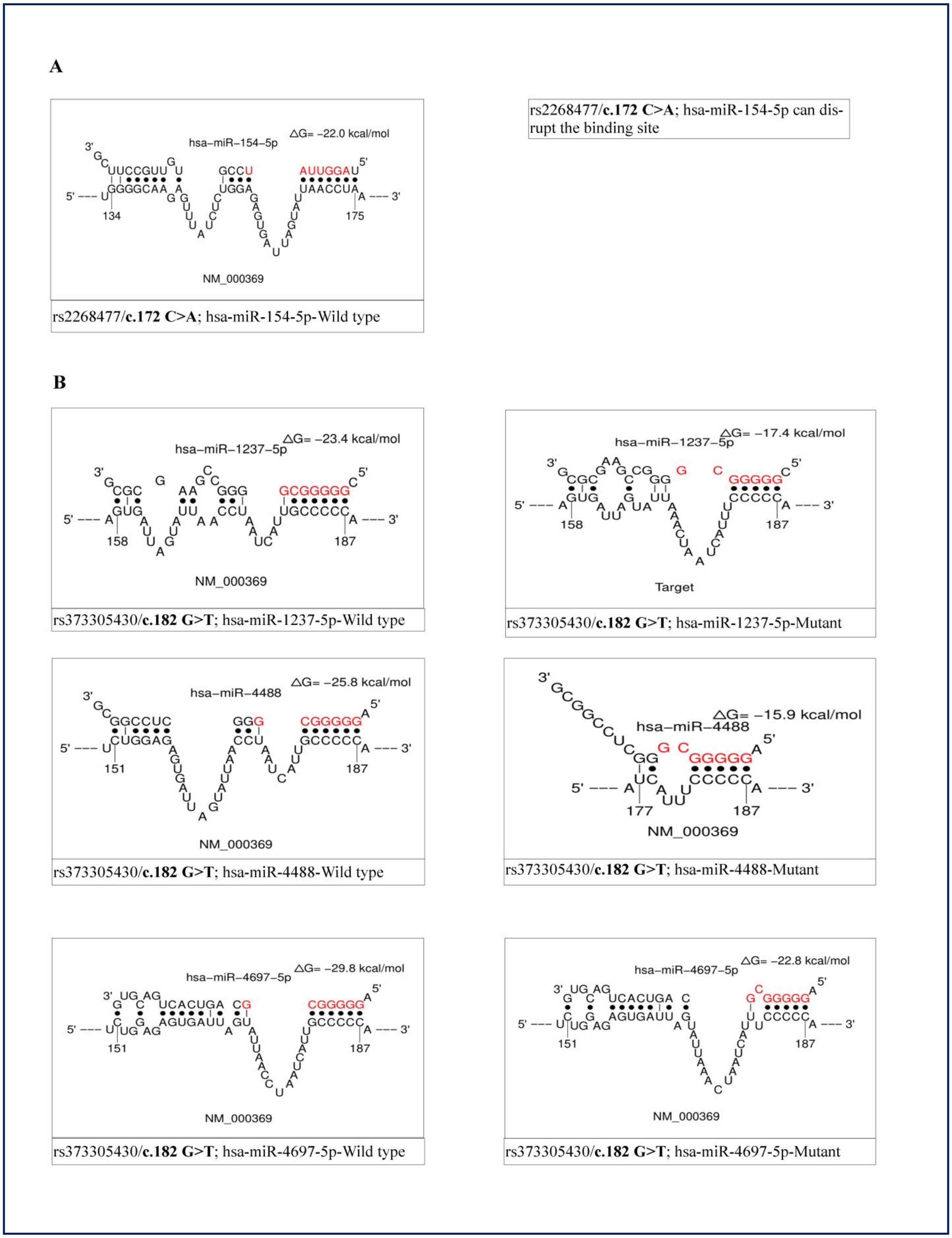

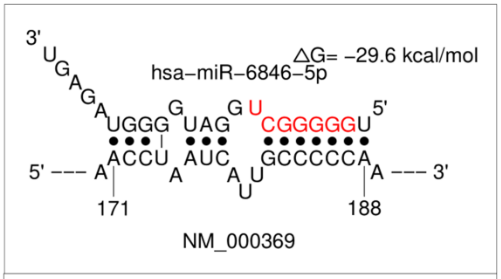

rs373305430/**c.182 G>T**; hsa-miR-6846-5p-Wild type

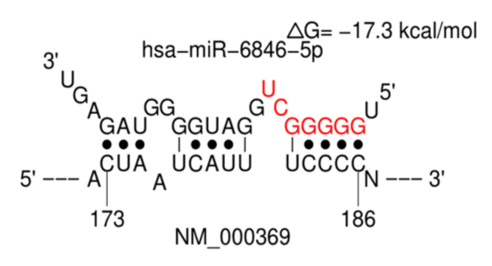

rs373305430/**c.182 G>T**; hsa-miR-6846-5p-Mutant

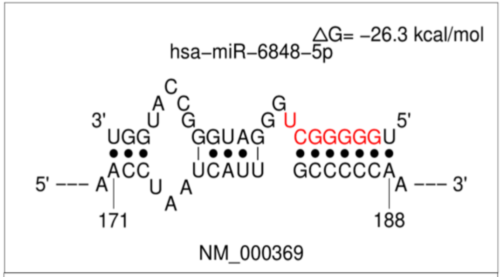

rs373305430/**c.182 G>T**; hsa-miR-6848-5p-Wild type

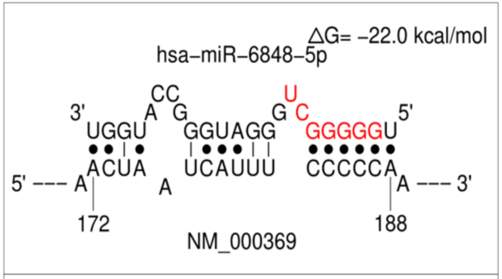

rs373305430/**c.182 G>T**; hsa-miR-6848-5p-Mutant

C

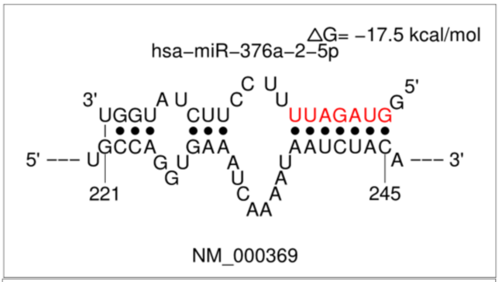

rs7144481/**c.245 C>T**; hsa-miR-376a-2-5p-Wild type

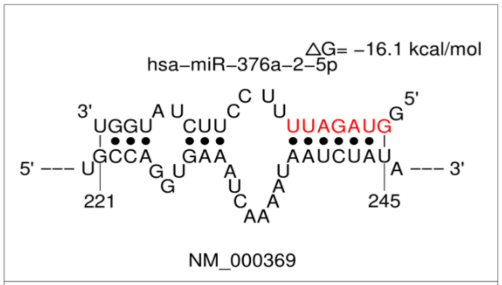

rs7144481/**c.245 C>T**; hsa-miR-376a-2-5p-Mutant

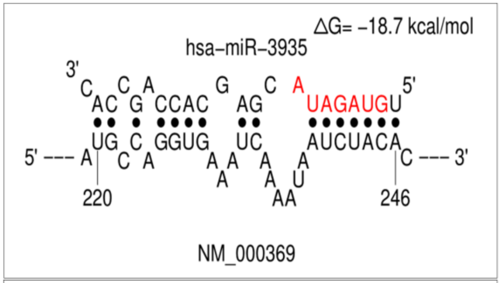

rs7144481/**c.245 C>T**; hsa-miR-3935-Wild type

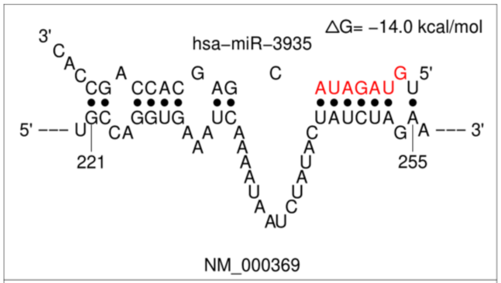

rs7144481/**c.245 C>T**; hsa-miR-3935-Mutant

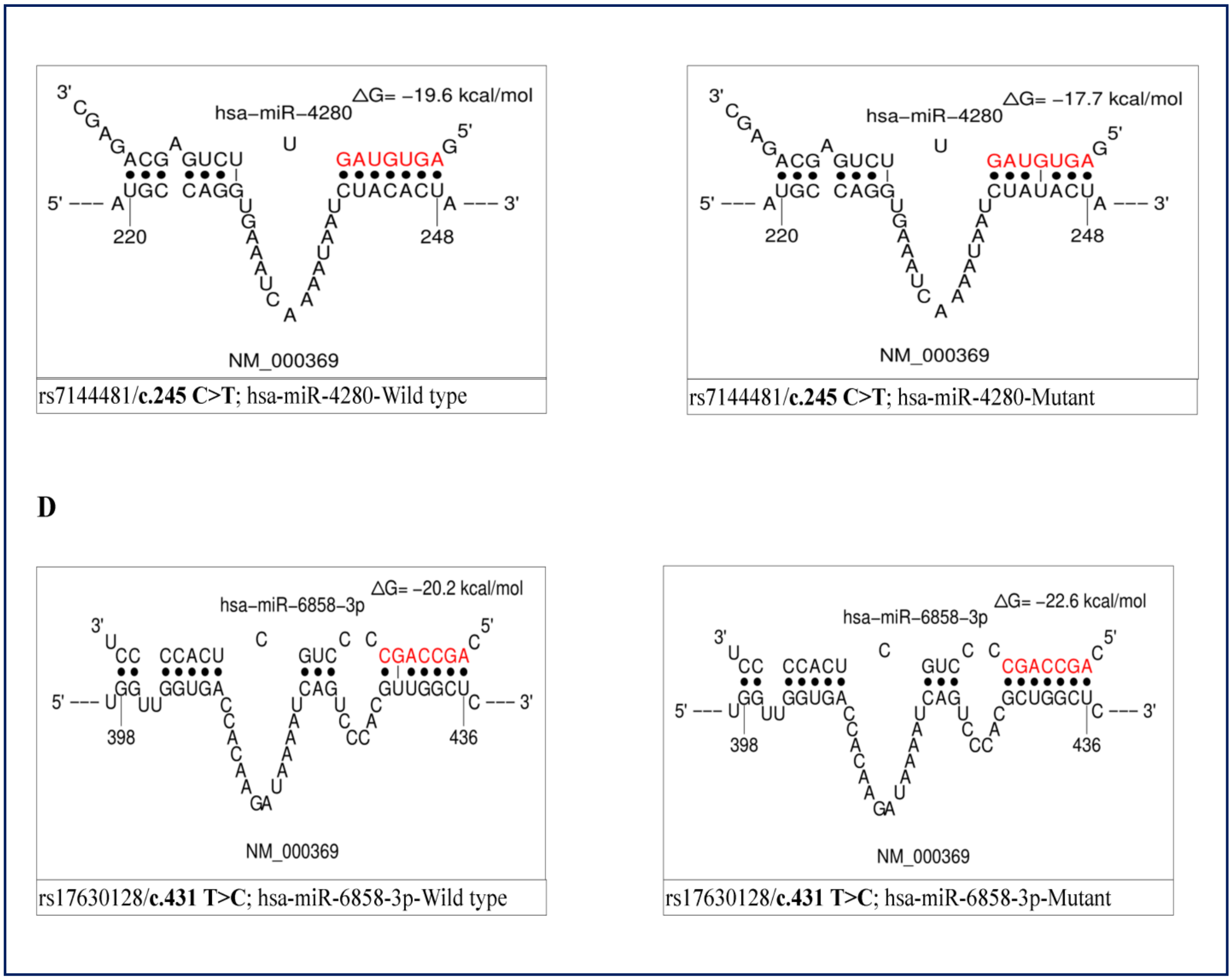

Supplementary Fig. 1. Schematic representation of the miRNA binding site on the 3'-UTR variants found in the *TSHR* gene. Hybrid diagram for a seed site (miRNA seed region in red) and hybrid diagram for a seedless (non-canonical) site. (A) rs2268477/c.172 C>A (hsa-miR-154-5p), which can destroy the binding site. (B) rs373305430/c.182 G>T (hsa-miR-1237-5p; hsa-miR-4488; hsa-miR-4697-5p; hsa-miR-6846-5p; hsa-miR-6848-5p). (C) rs7144481/c.245 C>T (hsa-miR-376a-2-5p; hsa-miR-3935; hsa-miR-4280). (D) rs17630128/c.431 T>C (hsa-miR-6858-3p).
